# Age‐related radiomorphometric changes on panoramic radiographs

**DOI:** 10.1002/cre2.375

**Published:** 2020-12-11

**Authors:** Yeon‐Hee Lee, Q‐Schick Auh, Yang‐Hyun Chun, Jung‐Sub An

**Affiliations:** ^1^ Department of Orofacial Pain and Oral Medicine Kyung Hee University Dental Hospital Seoul South Korea; ^2^ Department of Orthodontics Seoul National University Dental Hospital Seoul South Korea

**Keywords:** age estimation, first molar, mandibular canal, mental foramen, panoramic radiograph, pulp‐to‐tooth ratio

## Abstract

**Objectives:**

We aimed to analyse age‐related anatomical changes in teeth and mandibular structures using panoramic radiographs.

**Materials and Methods:**

We included 471 subjects aged 13–70 years (mean, 35.12 ± 18.72 years). Panoramic radiographs were used to record intraoral condition and radiomorphometric parameters. After grouping the subjects by age decade, descriptive statistics and analysis of variance were performed to assess age‐related patterns.

**Results:**

The number of missing teeth, endodontically treated teeth, full veneer crowns, and implant prosthesis increased with age (all *p* < .05). The prevalence of periodontitis significantly increased after the 40s and was the highest in the 60s (57.1%). The maxillary canine root was the longest in the 10s and 20s (*p* < .001). With age, the mandibular canal and mental foramen moved towards the alveolar bone crest, on the opposite side of the mandibular inferior border. The pulp area and pulp‐to‐tooth ratio of maxillary/mandibular first molars were significantly higher in the 10s and 20s than in other age groups (all *p* < .05).

**Conclusions:**

We provided comprehensive information on age‐related anatomical changes in teeth and mandibular structures based on panoramic radiographs. Various radiographic parameters showed specific changes with increasing age. Assessing these age‐related changes can be useful in determining an individual's age, and may aid in medico‐legal and forensic judgments.

## 1 INTRODUCTION

In forensics, estimating the age of living or dead individuals has become increasingly important, for reasons such as legal queries regarding immigration or refugee status, adoption, criminal offences, employment, and age verification (Juneja, Devi, Rakesh, & Juneja, 2014; Mathew et al., 2013). Methods for forensic analysis include radiographic evaluation of teeth to assess mineralisation, crown formation, eruption, root growth, and apical maturation (Ilayaraja et al., 2018; Mohammed et al., 2014). However, there is no consensus on the method for estimating age after the completion of permanent dentition. Furthermore, age estimation by radiographic methods in adults is not sufficiently accurate (Solheim, 1993).

Radiography is a vital, non‐invasive tool in forensic dentistry, and is used to uncover details that remain unknown during physical examinations (Juneja et al., 2014; Saxena, Sharma, & Gupta, 2010). Panoramic radiographs (PRs) are frequently used for radiographic evaluation in general dental practice, and have a great value in forensic science. PRs are cost‐effective, reproducible, and can be obtained with minimal radiation exposure (Devlin & Yuan, 2013). Furthermore, PRs have the advantage of simultaneously capturing multiple dental and surrounding anatomical structures. Thus, PRs are of crucial value in the forensic field.

Macroscopic dental changes, such as tooth loss and progression of periodontitis, are considered a normal part of the aging process. After teeth are fully formed, these gradual macroscopic changes usually appear with sustained wear and attrition of the teeth (Razak et al., 2014). Tooth surface loss is an irreversible process that progresses with age (Lambrechts, Braem, Vuylsteke‐Wauters, & Vanherle, 1989). The number of endodontically treated teeth and dental‐implant prostheses increases with age (Schwarz, 1996), and periodontitis is also more prevalent in older individuals (AlJehani, 2014). A hallmark of periodontitis progression is alveolar bone resorption (Hienz, Paliwal, & Ivanovski, 2015); therefore, in the elderly, periodontitis affects the clinical crown length.

Teeth are key indicators for dental age estimation, since changes in the teeth give a quick and rough idea of age (Rösing et al., 2007). Canines, the cornerstones of the dental arch, along with the first molars, are regarded as the most important teeth. Maxillary canines often have the longest root (Sapkota & Gupta, 2014) and suffer less wear than the other anterior teeth; thus, they are normally the oldest teeth in adults (Cameriere et al., 2007a). Of the posterior teeth, the first molars are selected for assessment, because they show fewer overlaps and superimposition issues on PRs than the premolars (Ilayaraja et al., 2018). The first molars are usually the first permanent teeth to erupt in the oral cavity; hence, they are most likely to exist in the oral cavity for the longest time and play a vital role in mastication. Furthermore, in the age estimation model, the standard error of the first molar (8.84 years) is less than that of the second molar (10.11 years; Shah & Venkatesh, 2016). Therefore, it is wise to use the first molar for a more accurate age estimation. It has been established, however, that age estimations using the pulp‐to‐tooth ratio in molars and anterior teeth are comparably accurate (Mathew et al., 2013).

The mandibular canal (MC) and mental foramen (MF) are vital structures in the mandible, as they have neurovascular bundles, such as the inferior alveolar neurovascular bundle, and the inferior alveolar or lingual artery (Juodzbalys, Wang, & Sabalys, 2010). The anatomical location of the MF can help determine the age and sex of an individual (Kanchan & Krishan, 2015). It is usually located more coronally than the MC. Mandibular changes primarily occur in the alveolar process, and changes in the basal bone occur throughout life (Afsar, Haas, Rossouw, & Wood, 1998). The mandible exhibits both age‐ and sex‐dependent morphological and anatomical changes (Okşayan et al., 2014).

Determining the age group of a subject is a key aspect in medico‐legal and forensic cases, as a range of ±10 years in forensic age prediction is considered acceptable (Babshet, Acharya, & Naikmasur, 2010; Mathew et al., 2013). Therefore, dividing subjects into age groups of 10‐year intervals and comparing radiographic changes across these age groups is forensically useful for estimating age. The aim of the present study was to investigate age‐related changes in oral conditions and PR image parameters. Consequently, we analyzed 14 parameters on PR images after creating groups of study subjects for each decade from the 10s to 60s. The parameters, including upper canine morphology, MC and MF position, and pulp‐tooth relationships of the first molars, have not been fully studied in Koreans. Our findings may help in estimating a person's age group, and provide information regarding the trends of various parameters easily found on PR images.

## 2 MATERIALS AND METHODS

The research protocol for the present study was reviewed in compliance with the Helsinki Declaration, and was approved by the Institutional Review Board of the Kyung Hee University Dental Hospital located in Seoul, Korea (KHD IRB). Informed consent was obtained from all participants.

### 2.1 Study population and design

The study population consisted of 471 subjects (222 males and 249 females) aged 13–70 years (mean age, 35.12 ± 18.72 years), who visited the Kyung Hee University Dental Hospital to receive dental care from 1 April 2017 to 31 May 2019, and received PRs. Based on power analysis with a significance (alpha error probability) level of .05 and a corresponding confidence level of 95% and power (1‐beta error probability) of 0.95 and sample size determination using G*Power (version 3.1.9.2, Franz Faul, Christian Albrechts‐Universitat, Kiel, Germany), we randomly selected 7% of the eligible subjects treated during the specified period.

The chronological age for each individual was calculated by subtracting the birth date listed on their official birth certificate from the date on which the radiographs were taken. Since an absolute error within ±10 years of the predicted age is considered acceptable in forensic age prediction (Cameriere et al., 2007b; Mathew et al., 2013), the subjects were divided into the following six groups: 139 subjects in their second decade (13–19 years), 82 in their third decade (20–29 years), 53 in their fourth decade (30–39 years), 64 in their fifth decade (40–49 years), 63 in their sixth decade (50–59 years), and 70 in their seventh decade (60–70 years).

We investigated age‐related changes in intraoral conditions and radiomorphometric parameters as seen on PRs. The inclusion criteria were as follows: all radiomorphometric parameters were clearly visible bilaterally on the PRs; the selected teeth (maxillary canines and first molars) had erupted fully; and the roots were fully formed. Subjects were excluded if they had mixed dentition; systemic disorders that could affect tooth maturation, eruption, or bone growth, such as amelogenesis or dentinogenesis imperfecta; any developmental, endocrine, or nutritional disorder; a history of any maxillofacial surgery or surgical procedure of the maxilla or mandible; or temporomandibular disorders.

PRs were taken for all subjects with the same panoramic dental imaging unit (Planmeca Promax; Planmeca OY, Helsinki, Finland), according to the manufacturer's instructions. The head was held in position using a chin rest and bite guide. The optimal image density and contrast were achieved using the following exposure settings: 84 kVp, 16 mA, and 16 s. The magnification factor was 1.20. PR data were saved as Digital Imaging and Communications in Medicine (DICOM) files, and a Picture Archiving Communication System (PACS; Infinitt Healthcare, Seoul, Korea) was used to analyse the DICOM data to establish reference lines and generate quantitative measurements.

### 2.2 Radiographic evaluation

The investigators (L.Y.‐H. and A.J.‐S.) independently performed visual assessments of the number of missing teeth, endodontically treated teeth, full veneer crowns of any material (i.e., gold, zirconia, porcelain fused to metal), and implant prostheses in each subject, and age‐related changes regarding these factors were analyzed. Since most healthy individuals have 28 permanent teeth by 13 years of age, 28 was considered the normal number of teeth for each subject. We evaluated the presence of periodontitis as a percentage, according to the age group. Periodontitis was diagnosed when ≥30% of the teeth had ≥5 mm distance from the alveolar bone level to the cementoenamel junction (CEJ; Renvert, Persson, & Persson, 2013). In some teeth, the alveolar bone crest heights were different between the labial and buccal sides; in such cases, the average of the two heights was set as the CEJ.

To test the repeatability of measurements, 30 subjects were randomly re‐evaluated 2 weeks after the initial measurements. Intraclass correlation coefficients for these analyses ranged from 0.92 to 0.99, indicating excellent reliability (Cronbach's *α*, 95% CI [0.92, 0.99], *p* < .001).

### 2.3 Evaluation of radiomorphometric parameters

**FIGURE 1:**
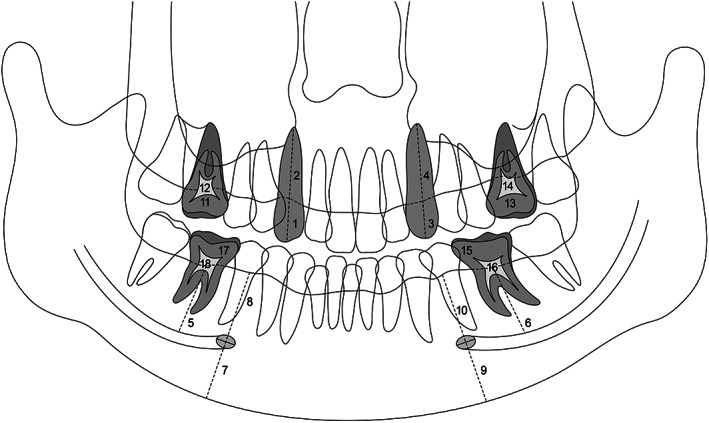
Schematic representation of 18 radiomorphometric parameters. Morphology of the upper canine represented by (1) crown length of the upper right maxillary canine, (2) root length of the upper right maxillary canine, (3) crown length of the upper left maxillary canine, and (4) root length of the upper left maxillary canine. Position of the mandibular canal (MC) represented by (5) mandibular bone thickness (MBT) above the right MC and (6) MBT above the left MC. Position of the mental foramen (MF) represented by (7) MBT below the right MF, (8) MBT above the right MF, (9) MBT below the left MF, and (10) MBT above the left MF. Pulp and tooth relationship of the upper and lower first molars represented by (11) tooth area of the upper right first molar, (12) pulp area of the upper right first molar, (13) tooth area of the upper left first molar, (14) pulp area of the upper left first molar, (15) tooth area of the lower left first molar, (16) pulp area of the lower left first molar, (17) tooth area of the lower right first molar, and (18) pulp area of the lower right first molar

We investigated 14 parameters, including five linear and four area measurements, on the PR images, and five parameters were derived from the original measurements. To assess the shape and length of the maxillary canines, the four parameters used were crown length, root length, tooth length, and crown‐to‐root ratio. To investigate the position of the MC, we examined the mandibular bone thickness (MBT) above the MC at the lower first molar. To identify the position of the MF, the following were assessed: MBT below the MF, MBT above the MF, and percent of the MBT below the MF. To identify the pulp‐tooth relationship of the first molars, tooth area, pulp area, and pulp‐to‐tooth ratio were assessed (Figure 1).

Each linear (in mm) and area (cm^2^) measurement was taken bilaterally, thus accounting to 18 measurements for each individual, using the PACS software. Additionally, five parameters were derived from these measurements, one from the sum of the two length measurements, and four from the ratios of the two measurements.

#### 2.3.1 Morphology of the upper canine

The maxillary canine crown length (the distance from the incisal edge to the alveolar bone crest) and root length (the distance from the alveolar bone crest to the apex) were measured along the longitudinal axis, which is the line extending inciso‐cervically from the crown tip to the root apex. The total length (the distance from the incisal edge to the apex) of the maxillary canine was measured by adding these two measurements (Figure 1).

#### 2.3.2 Position of the MC

At the centre of the mandibular first molar, two points forming the shortest distance from the alveolar bone crest to the inferior mandibular border were located, and a line connecting these two points was drawn. Next, a tangential line was drawn at the junction between this line and the superior border of the MC. The MBT above the MC was then obtained by measuring the distance from the superior border of the MC to the alveolar bone crest (Figure 1).

#### 2.3.3 Position of the MF

A tangential line was drawn along the inferior border of the mandible below the MF, and a perpendicular line passing through the centre of the MF was then drawn. The MBT below and above the MF were obtained by measuring the distance from the centre of the MF to the inferior border of the mandible and to the alveolar bone crest on the perpendicular line, respectively. To determine the vertical change of the MF relative to MBT, the percentage of the MBT below the MF was determined by calculating the ratio of MBT below the MF to MBT (Figure 1).

#### 2.3.4 Pulp‐tooth relationship of the first molars

The tooth and pulp areas of the maxillary and mandibular first molars (#16, 26, 36, and 46) were measured using the PACS software. The pulp‐to‐tooth ratio was determined by calculating the ratio of the two areas (Figure 1).

### 2.4 Statistical analysis

The means and their associated standard deviations were calculated for continuous variables, and differences were analyzed using two‐way factorial analysis of variance with respect to both age group and sex. Frequencies and percentages were calculated for categorical variables and were compared between groups using the chi‐squared test. Pearson correlations between age and radiomorphometric parameters were calculated. Intraclass correlation coefficients were used to measure intra‐rater agreement. IBM SPSS Statistics software (version 22.0; IBM, Armonk, NY) was used for all statistical analyses. A two‐tailed *p*‐value of less than .05 was considered statistically significant.

## 3 RESULTS

### 3.1 Demographics and results from radiographic inspection

**FIGURE 2:**
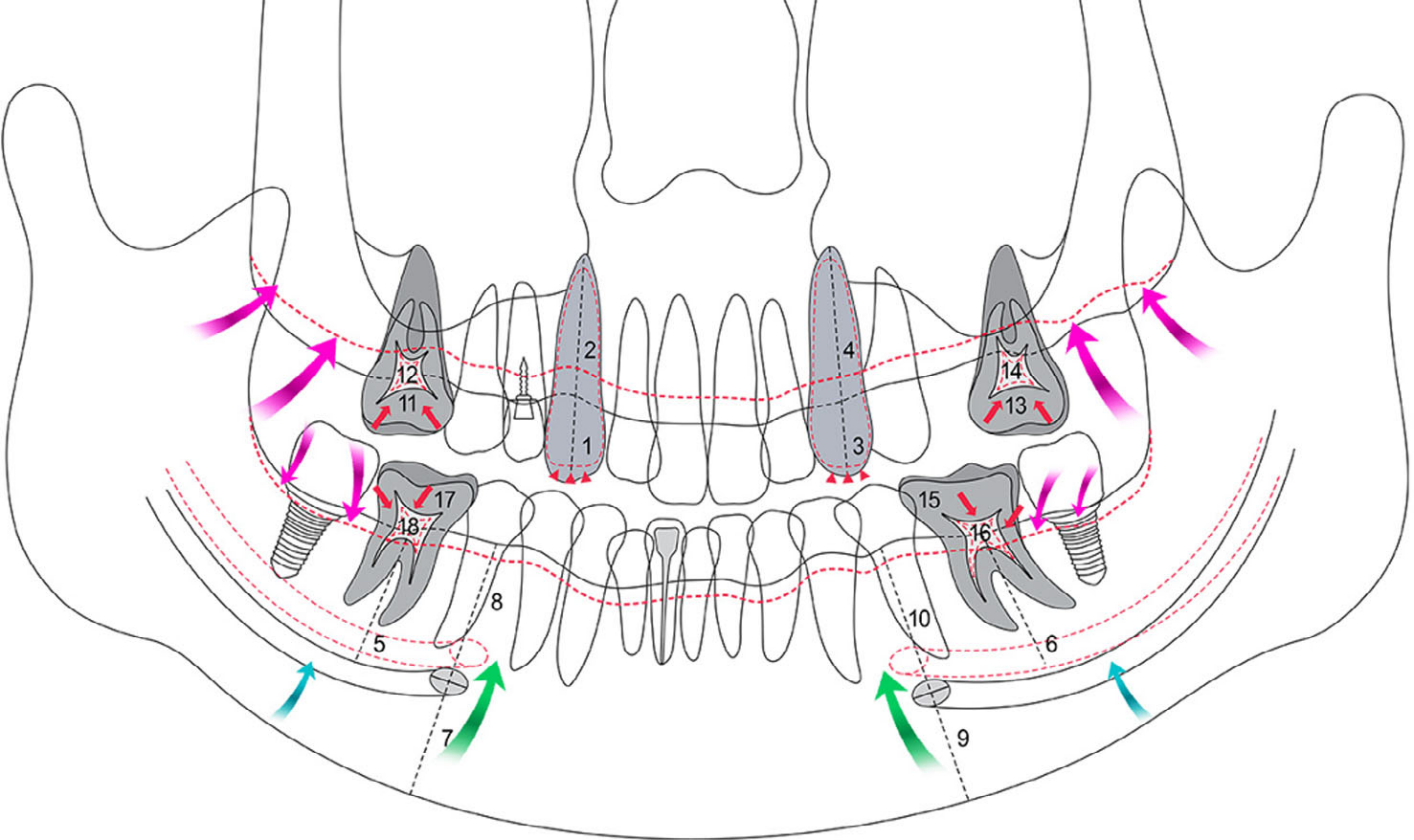
Schematic diagram of radiomorphometric parameters by age

Sex distribution did not vary significantly by age group (*p* = .400, Table 1). The number of treated and missing teeth increased with age (Figure 2). The number of endodontically treated teeth was significantly higher in the 60s than in the 10s and 20s (*p* < .001). The number of endodontically treated teeth (3.27 ± 9.36) and full veneer crown restorations (8.29 ± 6.32) was the highest in the 60s, and both increased with age. The number of treated teeth did not differ by sex. The 60s age group showed a statistically significant increase in the number of missing teeth compared to the other age groups (*p* < .001). The number of missing teeth did not differ by sex (*p* = .101; Table 1).

**TABLE 1:** Age and intraoral condition of subjects according to age group

Variable	Age groups						Significance^a^			Multiple comparisons
	10s	20s	30s	40s	50s	60s	Sex	Age groups	Interaction	
	(*n* = 139)	(*n* = 82)	(*n* = 53)	(*n* = 64)	(*n* = 63)	(*n* = 70)				
Age (years)	15.09 ± 2.77	21.76 ± 1.06	34.62 ± 3.01	45.20 ± 2.98	53.16 ± 1.84	65.50 ± 2.28	< .001	.807	.331	Female = male
										10s < 20s < 30s < 40s < 50s < 60s
Number of:										
Endodontically treated teeth	0.12 ± 0.58	0.29 ± 0.79	1.32 ± 2.16	1.56 ± 1.91	1.43 ± 1.85	3.27 ± 9.36	.595	< .001	.417	Female = male
										10s = 20s < 60s
Full veneer crowns	0.20 ± 1.02	0.26 ± 0.87	2.21 ± 3.78	2.82 ± 3.15	3.97 ± 4.36	8.29 ± 6.32	.093	< .001	.532	Female = male
										10s = 20s < 40s = 50s < 60s, 10s < 30s < 60s
Missing teeth	0.68 ± 1.37	1.05 ± 1.66	1.08 ± 2.66	1.05 ± 1.91	1.40 ± 3.00	3.50 ± 5.95	.101	< .001	.657	Female = male
										10s = 20s = 30s = 40s = 50s < 60s
Implant prostheses	0.00 ± 0.00	0.10 ± 0.51	0.43 ± 1.20	0.58 ± 1.41	1.19 ± 2.61	2.41 ± 3.31	< .001	< .001	.004	Female: 10s = 20s = 30s = 40s < 60s, 10s < 50s
										Male: 10s = 20s = 30s = 40s = 50s < 60s

^a^ Two‐way analysis of variance was used to determine significant differences between the groups.

The number of implant prostheses differed by sex. Females had significantly more implant prostheses in their 60s than in their 10s–40s, and more in their 50s than in their 10s. Males in their 60s had significantly more implant prostheses than the other age groups; however, there was no significant difference between the other age groups. The average number of implant prostheses was <1.0 in the 10s–40s, but was higher in the 60s (2.41 ± 3.31, Table 1).

Periodontitis was not observed in the 10s and 20s; however, the prevalence increased with age (9.4, 25, 36.5, and 57.14% in the 30s, 40s, 50s, and 60s, respectively). The prevalence of periodontitis did not differ by sex in each age group (Table 2).

**TABLE 2:** Sex and periodontitis rates of the subjects according to age group

	Age groups						
	10s (*n* = 139)	20s (*n* = 82)	30s (*n* = 53)	40s (*n* = 64)	50s (*n* = 63)	60s (*n* = 70)	Significance
Sex							
Female (% of age group)	86 (61.9)	48 (58.5)	25 (47.2)	32 (50.0)	33 (52.4)	38 (54.3)	Chi‐squared statistic for homogeneity of sex: *p* = .400
Male (% of age group)	53 (38.1)	34 (41.5)	28 (52.8)	32 (50.0)	30 (47.6)	32 (45.7)	
Periodontitis (% of age group)	0 (0.0)	0 (0.0)	5 (9.4)	16 (25.0)	23 (36.5)	40 (57.1)	Chi‐squared statistic for trend: *p* < .001
Female (% of age group)	0 (0.0)	0 (0.0)	1 (20.0)	6 (37.5)	6 (26.1)	19 (38.1)	Homogeneity of sex in periodontitis patients: *p* = .343
Male (% of age group)	0 (0.0)	0 (0.0)	4 (80.0)	10 (62.5)	17 (73.9)	21 (61.9)	

### 3.2 Age‐related differences in radiomorphometric parameters

Table 3 shows the radiomorphometric parameters, including the morphology of the upper canine, position of the MC and MF, and pulp‐tooth relationship of the first molars, based on sex and side.

**TABLE 3:** Differences in radiomorphometric parameters with respect to sex and side

Variable	Female		Male		Significance^a^			Comparisons
	Right	Left	Right	Left	Sex	Side	Interaction	
*Morphology of the upper canine*								
	(*n* = 255)	(*n* = 254)	(*n* = 199)	(*n* = 199)				
Crown length (mm)	9.65 ± 1.90	9.55 ± 1.87	10.19 ± 2.24	10.31 ± 2.64	<0.001	0.933	0.453	Female < male, right = left
Root length (mm)	17.21 ± 3.04	17.23 ± 3.26	18.08 ± 3.33	18.19 ± 3.32	<0.001	0.752	0.844	Female < male, right = left
Tooth length (mm)	26.86 ± 3.74	26.79 ± 3.71	28.27 ± 3.91	28.50 ± 3.93	<0.001	0.753	0.555	Female < male, right = left
Crown‐to‐root ratio (%)	57.77 ± 15.79	58.09 ± 20.93	58.64 ± 19.10	59.38 ± 23.06	0.413	0.689	0.873	Female = male, right = left
*Position of the mandibular canal*								
	(*n* = 240)	(*n* = 233)	(*n* = 180)	(*n* = 174)				
MBT above the MC at the lower first molar (mm)	22.84 ± 3.82	23.13 ± 4.12	25.04 ± 4.41	25.07 ± 4.52	<0.001	0.584	0.676	Female < male, right = left
*Position of the mental foramen*								
	(*n* = 262)	(*n* = 262)	(*n* = 209)	(*n* = 209)				
MBT below the MF (mm)	14.18 ± 2.43	14.32 ± 2.41	15.89 ± 2.68	15.73 ± 2.68	<0.001	0.952	0.380	Female < male, right = left
MBT above the MF (mm)	19.51 ± 3.90	19.12 ± 3.49	20.68 ± 3.94	20.48 ± 3.71	<0.001	0.227	0.700	Female < male, right = left
Percent of the MBT below the MF (%)	42.31 ± 5.79	42.94 ± 5.15	43.62 ± 5.55	43.57 ± 5.50	0.007	0.422	0.349	Female < male, right = left
*Pulp and tooth relationship of the upper first molars*								
	(*n* = 238)	(*n* = 237)	(*n* = 174)	(*n* = 175)				
Tooth area (cm^2^)	1.72 ± 0.43	1.75 ± 0.44	1.86 ± 0.45	1.86 ± 0.41	<0.001	0.668	0.645	Female < male, right = left
Pulp area (cm^2^)	0.11 ± 0.05	0.10 ± 0.05	0.11 ± 0.05	0.11 ± 0.05	0.190	0.192	0.933	Female = male, right = left
Pulp‐to‐tooth ratio (%)	6.42 ± 2.76	6.05 ± 2.67	6.24 ± 2.43	6.01 ± 2.70	0.568	0.114	0.709	Female = male, right = left
*Pulp and tooth relationship of the lower first molars*								
	(*n* = 240)	(*n* = 228)	(*n* = 177)	(*n* = 175)				
Tooth area (cm^2^)	1.89 ± 0.45	1.95 ± 0.43	2.01 ± 0.46	2.04 ± 0.44	0.001	0.155	0.568	Female < male, right = left
Pulp area (cm^2^)	0.17 ± 0.07	0.18 ± 0.08	0.17 ± 0.08	0.18 ± 0.08	0.580	0.338	0.746	Female = male, right = left
Pulp‐to‐tooth ratio (%)	9.10 ± 3.49	9.23 ± 3.90	8.76 ± 3.89	8.89 ± 3.87	0.200	0.636	0.988	Female = male, right = left

Abbreviations: MBT, mandibular bone thickness; MC, mandibular canal; MF, mental foramen.

^a^ Two‐way analysis of variance was used to determine significant differences between the groups.

The upper canine crown, root, and tooth lengths were significantly greater in males than in females. However, the crown‐to‐tooth ratio did not differ significantly by sex. Parameters indicating the relative position of MC or MF for MBT were also greater in males than in females. The tooth area of all first molars was significantly greater in males than in females, but other parameters, including the pulp area and the pulp‐to‐tooth ratio, did not differ by sex.

**FIGURE 3:**
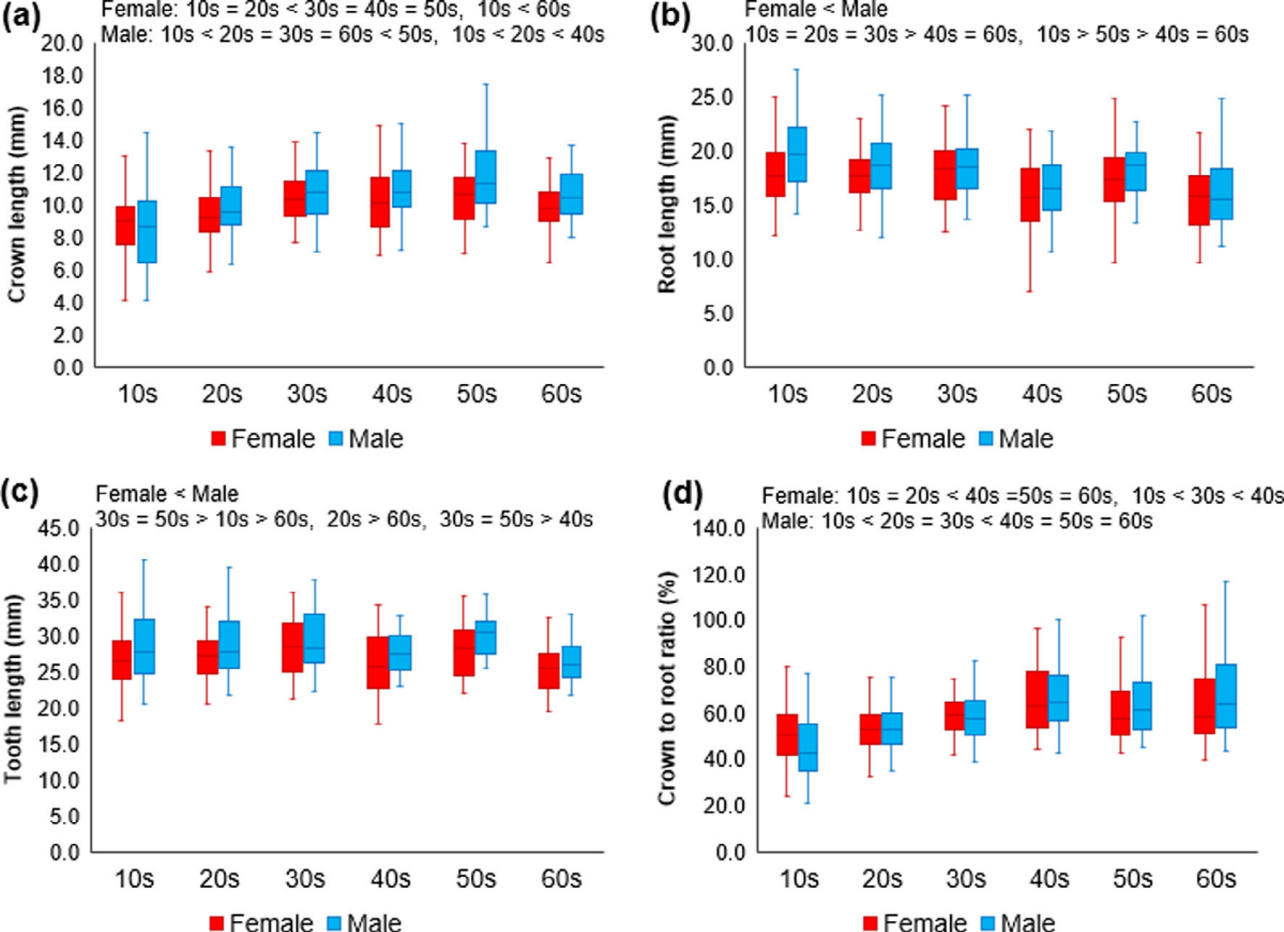
Morphology of the upper canine according to age group. (a) Crown length (mm), (b) root length (mm), (c) tooth length (mm), and (d) crown‐to‐root ratio (%)

**FIGURE 4:**
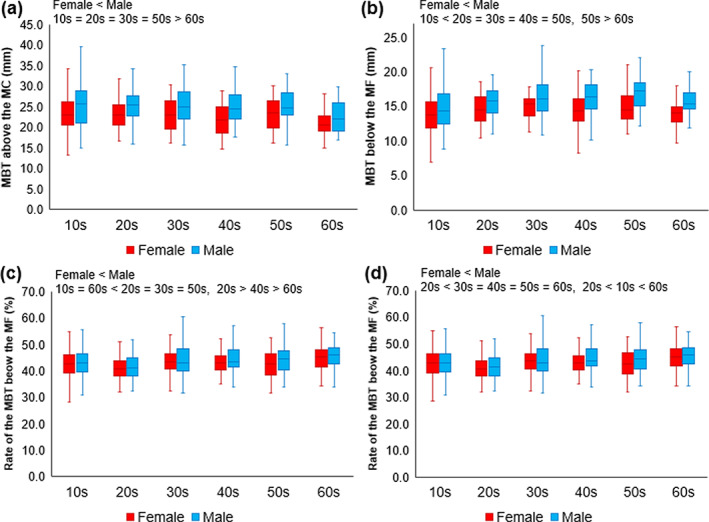
Position of the mandibular canal and mental foramen according to age group. (a) Mandibular bone thickness above the mandibular canal (mm), (b) mandibular bone thickness below the mental foramen (mm), (c) mandibular bone thickness above the mental foramen (mm), and (d) percent of the mandibular bone thickness below the mental foramen (%)

**FIGURE 5:**
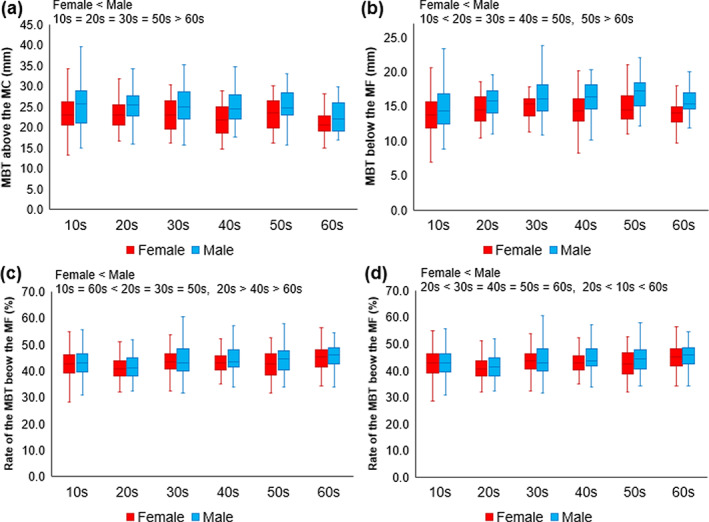
Pulp‐tooth relationship of the first molars according to age group. (a) Tooth area of the upper first molar (cm^2^), (b) pulp area of the upper first molar (cm^2^), (c) pulp‐to‐tooth ratio of the upper first molar (%), (d) tooth area of the lower first molar (cm^2^), (e) pulp area of the lower first molar (cm^2^), and (f) pulp‐to‐tooth ratio of the lower first molar (%)

Since none of the parameters differed significantly between the left and right sides, each was analyzed by age group and sex without distinguishing between the sides (Figures 3, 4, 5. Full data in Table S1).

### 3.3 Morphology of the upper canine

The crown length of the upper canine increased with age, but the trends varied by sex. Among females, it was significantly greater in the 30s, 40s, and 50s than in the 10s and 20s, and significantly greater in the 60s than in the 10s. Among males, crown length was the greatest in the 50s, and greater in the 20s, 30s, and 60s than in the 10s. Crown length also increased significantly with increasing age among males in their 10s, 20s, and 40s (Figure 3).

Upper canine root length was greater in males than in females, and decreased with age. Those in their 40s and 60s had shorter root lengths than those in their 10s, 20s, and 30s. Additionally, root length was significantly shorter in the 40s and 60s than in the 10s (Figure 3). Upper canine tooth length was also greater in males than in females, although it did not have a consistently linear relationship with age. Tooth length was greater among those in their 30s and 50s than among those in other age groups (Figure 4).

Crown‐to‐root ratio increased with age, and there was a statistically significant interaction between age groups and sex. Among females, the ratio was greater in the 40s, 50s, and 60s than in the 10s and 20s, and increased significantly across the 10s, 30s, and 40s. Among males, the ratio was the greatest in the 40s, 50s, and 60s, followed by in the 20s and 30s, and was the smallest in the 10s (Figure 3).

### 3.4 Position of the MC and MF

The MBT above and below the MF, and the percentage of the MBT below the MF were both greater in males than in females, but the trends varied by age group (Figure 4). The MBT above the MF was significantly thicker among those in their 20s, 30s, and 50s than in those in their 10s and 60s, and statistically decreased in the order of the 20s, 40s, and 60s. The MBT below the MF was thicker in the 20s‐50s than in the 10s, and in the 50s than in the 60s. The percentage of the MBT below the MF was significantly greater in the 30s‐60s than in the 20s, and increased in the order of the 20s, 10s, and 60s (Figure 4).

### 3.5 Pulp‐tooth relationship of the first molars

The tooth and pulp areas and the pulp‐to‐tooth ratio of the first molars did not differ by sex. One exception to this was the tooth area of the lower first molar, which was significantly greater in males than in females. The tooth area of the upper first molar did not vary by age group, but the tooth area of the lower first molar was significantly smaller in the 60s than in the other age groups, except the 40s (Figure 5).

The pulp area decreased with increasing age. This trend was more pronounced in the lower first molar than in the upper. The pulp area of the upper first molar was the largest in the 10s, and significantly smaller in the 40s and 60s. In the lower first molar, the pulp area was the largest in the 10s, followed by the 30s, and was the smallest in the 40s–60s. It did not differ significantly between the 20s and 30s, and was larger in these age groups than in the 40s, 50s, and 60s (Figure 5).

The pulp‐to‐tooth ratio also decreased with age, and this trend was more pronounced in the lower first molar than in the upper. In the upper first molar, pulp‐to‐tooth ratios were significantly lower in the 30s, 40s, 50s, and 60s than in the 10s. In the lower first molar, the pulp‐to‐tooth ratio was the highest in the 10s and 20s, significantly lower in the 30s than in the 10s, and significantly lower in the 50s than in the 30s.

### 3.6 Correlation between age and radiomorphometric parameters

Results of the Pearson correlation analysis demonstrated that radiomorphometric parameters were significantly correlated with age and showed similar trends in both sexes (Table 4). For both sexes, age was positively correlated with the crown length and crown‐to‐root ratio of the upper canine, and the percentage of the MBT below the MF. Age was negatively correlated with the root length of the upper canine and the pulp area and pulp‐to‐tooth ratio of the first molars (Table 4). However, age was positively correlated with the MBT above the MC in the lower first molar in females only, and with the MBT below the MF in males only. Conversely, age was negatively correlated with the tooth area of the upper first molar in females only (Table 4).

**TABLE 4:** Correlation between age and radiomorphometric parameters

Variable	Correlation with age		
	Female		Male
*Morphology of the upper canine*			
Crown length (mm)	0.292**		0.404**	
Root length (mm)	−0.251**		−0.345**
Tooth length (mm)	−0.065		−0.040
Crown‐to‐root ratio (%)	0.367**		0.471**
*Position of the mandibular canal*			
MBT above the MC at the lower first molar (mm)	−0.125**		−0.104
*Position of the mental foramen*			
MBT below the MF (mm)	0.074		0.222**
MBT above the MF (mm)	−0.066		−0.016
Percent of the MBT below the MF (%)	0.130**		0.177**
*Pulp and tooth relationship of the upper first molars*			
Tooth area (cm^2^)	**−0.103** *		−0.022
Pulp area (cm^2^)	−0.217**		−0.196**
Pulp‐to‐tooth ratio (%)	−0.212**		−0.206**
*Pulp and tooth relationship of the lower first molars*			
Tooth area (cm^2^)	−0.080		−0.037
Pulp area (cm^2^)	−0.385**		−0.394**
Pulp‐to‐tooth ratio (%)	−0.381**		−0.424**

Abbreviations: MBT, mandibular bone thickness; MC, mandibular canal; MF, mental foramen.

* Pearson correlation is significant at .05. **Pearson correlation is significant at .01.

## 4 DISCUSSION

Determining an individual's age is a crucial component of both forensic dentistry and forensic science. Estimating the age of living individuals, rather than solely cadavers, has now assumed a greater role in solving civil and criminal judicial problems (Ilayaraja et al., 2018). Although several methods of age estimation have been suggested, none is completely applicable for all ages (Ilayaraja et al., 2018; Mohammed et al., 2014). After the complete eruption of the permanent dentition, age estimation methods based on dental morphology using X‐rays are not accurate (Solheim, 1993). Therefore, we have attempted to devise a comprehensive method that can be used across 10–60 years of age, and to investigate the radiomorphometric parameters necessary for forensic age estimation using PRs, which are routine radiographs and have the advantage of being easy to obtain.

The results of the present study indicate that the number of treated teeth, including endodontics and full veneer crowns, increases progressively with age. Since cumulative periodontal destruction exposes dental roots, there is an increased risk of caries among the elderly (Lopez, Smith, Gostemeyer, & Schwendicke, 2017). Throughout life, the teeth and other surrounding oral tissues experience both mechanical and chemical wear and breakage from daily food intake and mastication (Schwarz, 1996; Van't Spijker et al., 2009). Therefore, as age increases, the number of treated teeth increases, which is natural and likely inevitable.

Tooth loss is considered a hallmark of late adulthood. The present study found that the number of teeth lost significantly increased in the 60s (3.50 ± 5.95). A previous study noted a mean tooth loss rate of 0.6–1.5 teeth per 10 years (Copeland, Krall, Brown, Garcia, & Streckfus, 2004). Tooth loss is a multi‐factorial process involving dental caries, periodontal disease, general health status, and a variety of socio‐environmental factors, including educational level, access to care, socio‐economic status, and insurance status (Tiwari, Scarbro, Bryant, & Puma, 2016). Thus, tooth loss reflects the lifelong cumulative effects of both disease and social factors (Copeland et al., 2004). Additionally, the number of teeth in the mandible is directly proportional to mandibular height, and tooth loss is correlated with a decrease in alveolar bone level (Ozturk et al., 2013), a relationship which can be bidirectional.

As the number of missing teeth increases in an aging population, implant prostheses can offer an effective and predictable course of treatment, replacing traditional restorations (Gowd, Shankar, Ranjan, & Singh, 2017). There is an increasing demand among the elderly for dental implants to replace missing teeth. In the present study, the number of implant prostheses averaged <1.0 in each age group from the 10s to the 40s, and increased rapidly in the 60s (2.41 ± 3.31). One cohort study reported that approximately 3% of older adults had dental implants, 49% of whom were aged 65 years or older (Sato, Kitagawa, & Isobe, 2018). The prevalence of edentulism increases with age (Dogan & Gokalp, 2012). However, the number of natural teeth retained in older adults is increasing, resulting in a lower proportion of patients needing prostheses to replace missing teeth (Sato et al., 2018). Therefore, the relationship between the number of implant prostheses and age may vary over time, and more follow‐up studies are needed.

Periodontitis is one of the factors known to contribute to tooth loss. In the present study, the prevalence of periodontitis increased with age; it increased significantly and sharply after the 40s, and peaked in the 60s (57.14%), when the number of missing teeth also increases. In people aged 65 years and older, the prevalence of periodontitis is over 50%, and is independent of location or race (Demmer & Papapanou, 2010). However, depending upon the criteria used to define periodontitis, the prevalence ranges from 14 to 65% (Costa et al., 2009; Demmer & Papapanou, 2010). Additionally, the rate of prevalence increases with lower socio‐economic status and poor oral health (Vargas, Yellowitz, & Hayes, 2003). Unfortunately, the present study did not account for participants' oral hygiene or socio‐economic status. Therefore, studies with a more detailed design are needed to confirm our results.

Macroscopic changes in the teeth are considered part of the normal aging process. Typical age‐related changes in the tooth macrostructure include wear, resection, and root resorption (Ilayaraja et al., 2018). In the present study, decreased root length and increased crown length of the canines were observed with age, which are considered to be key teeth for personal identification (Yuwanati, Karia, & Yuwanati, 2012). Tooth length is considered important in determining age, because crown length above the level of the alveolar bone decreases with age (Jayawardena, Abesundara, Nanayakkara, & Chandrasekara, 2009). Gustafson (1950) described morphological changes, such as attrition and root resorption, that occur with increased age. Gustafson's method has the maximum accuracy around ages 40–50 years, and has been widely used to date (Solheim & Vonen, 2006). However, the fatal flaw with this method is that it cannot be used in a living person, only with posthumous tooth extraction. Moreover, these methods may be unacceptable for cultural, ethical, or scientific reasons, so our methods may be complementary or alternative.

Skeletal size likely contributes, at least moderately, towards the accuracy of sex determination. Several parameters related to both canine morphology and tooth area of the first molars were larger in males than females. One reason for this is the relatively larger skeletal framework of males compared to females (Ozturk et al., 2013). Canines, more than any other teeth, have been found to genetically exhibit the greatest sexual dimorphism (Garn, Lewis, Swindler, & Kerewsky, 1967; Yuwanati et al., 2012). Hormones and nutrition both contribute towards the craniofacial morphological differences between sexes (Seeman, 2003; Nieves et al., 2005; Kasat, Karjodkar, & Vaz, 2010). Taking these differences into account, it seems that prioritizing sex classification and then examining other characteristics of the jaw increases the efficiency of age estimation. It was worth noting that the 14 radiomorphometric parameters we compared did not differ between the left and right sides, as seen in previous studies (Phulari, Rathore, Talegaon, & Jariwala, 2017).

Vital structures in the mandible, such as the MC and the MF, have been examined in for their value in age estimation. The results of our present study indicated that the MC and MF both migrate upwards towards the alveolar bone crest as age increases. Mandibular remodelling also occurs throughout life, based on age, gender, and dental status (Ashkenazi, Taubman, & Gavish, 2011; Bhardwaj, Kumar, & Mohan, 2014). The MC gradually approaches the alveolar border, based on the degree of bone resorption (Bhardwaj et al., 2014; Kanchan & Krishan, 2015). As relative vertical movement of the MF occurs, it then lies closer to the inferior border of the mandible, and moves upwards closer to the alveolar border in old age due to tooth loss and bone resorption (Bhardwaj et al., 2014; Kanchan & Krishan, 2015). The location and size of the MF both vary with sex and race (Apinhasmit, Methathrathip, Chompoopong, & Sangvichien, 2006). In the field of forensics, the MF is one of the anatomical landmarks used for the identification of human remains, and various forensic anthropological studies have been conducted on the shape, size and position of the MF (Apinhasmit et al., 2006). Nevertheless, anatomical variations that may occur with respect to the location of the MF and MC should always be taken into account.

In the present study, the pulp area and pulp‐to‐tooth ratio of the first molars were significantly lower in the 60s compared to the 50s, and the pulp‐to‐tooth ratio was negatively correlated with age. An examination of extracted teeth showed that the size of the dental pulp cavity is reduced due to secondary dentin deposition Kvaal, Kolltveit, Thomsen, and Solheim (1995), and the measurements of this reduction can be used as an indicator of age. Cameriere, Ferrante, and Cingolani (2004) conducted a preliminary study evaluating variations in pulp‐to‐tooth ratio of molars, which appeared to be a promising indicator of age on orthopantomograms. Radiographic age estimation in children using teeth relies on their developmental stage, whereas in adults, the continuous deposition of secondary dentin along the walls of the pulp chamber leads to a decrease in its area, volume, and size, which can aid in the estimation of age (Abou Neel et al., 2016).

The present study has some limitations. It is not possible to use our method for age estimation in children and adolescents with primary or mixed dentitions. Additionally, our method did not estimate the exact age, but only identified information that can be used to estimate the age group by decade. This study included only Korean subjects, and caution should be executed while extrapolating the results to people of other races. Future research studies using larger samples are needed for a clearer and more meaningful interpretation of these results.

In the present study, we sought to provide comprehensive information for age group estimation based on PRs, and to determine the trends for various radiomorphometric parameters by age. Our comprehensive, age‐based survey using routinely taken PRs is likely to be useful in the field of forensic medicine in the future.

## CONFLICT OF INTEREST

The authors declare no conflict of interest.

## AUTHOR CONTRIBUTIONS

Conception and design: Yeon‐Hee Lee and Jung‐Sub An. Acquisition of data: Yeon‐Hee Lee and Q‐Schick Auh, Yang‐Hyun Chun. Analysis and interpretation of data: Yeon‐Hee Lee, Q‐Schick Auh, and Jung‐Sub An. Drafting the manuscript: Yeon‐Hee Lee. Revising the manuscript for intellectual content: Yeon‐Hee Lee. Final approval of the completed manuscript: Yeon‐Hee Lee, Q‐Schick Auh, and Jung‐Sub An.

## Supporting information

**Table S1** STROBE Statement—checklist of items that should be included in reports of observational studies

## Data Availability

The datasets generated during and/or analysed during the current study are available from the corresponding author on reasonable request.
